# The neurological and cognitive consequences of hyperthermia

**DOI:** 10.1186/s13054-016-1376-4

**Published:** 2016-07-14

**Authors:** Edward James Walter, Mike Carraretto

**Affiliations:** Department of Intensive Care Medicine, Royal Surrey County Hospital, Egerton Road, Guildford, Surrey GU2 7XX UK

**Keywords:** Brain injury, Fever, Hyperthermia, Cognition, Cerebellum

## Abstract

An elevated temperature has many aetiologies, both infective and non-infective, and while the fever of sepsis probably confers benefit, there is increasing evidence that the central nervous system is particularly vulnerable to damage from hyperthermia. A single episode of hyperthermia may cause short-term neurological and cognitive dysfunction, which may be prolonged or become permanent. The cerebellum is particularly intolerant to the effects of heat. Hyperthermia in the presence of acute brain injury worsens outcome. The thermotoxicity involved occurs via cellular, local, and systemic mechanisms. This article reviews both the cognitive and neurological consequences and examines the mechanisms of cerebral damage caused by high temperature.

## Background

An elevated temperature by whatever cause, infective or non-infective, affects many organ systems of the body, sometimes with damage which is irreversible, and may lead to death. A temperature of 37.5 °C or greater at any point during an admission to the intensive care unit (ICU) trends towards a worse outcome, and becomes significant at temperatures greater than 38.5 °C [1]. There is emerging evidence that the central nervous system is especially vulnerable to hyperthermia, particularly if prolonged or excessive. Only in sepsis is there a likelihood that some levels of temperature elevation may afford teleological benefits leading to a survival benefit, but once the temperature rises above 40 °C it is once again associated with a worse outcome [1].

Non-infective causes of hyperthermia include heat illness and drug-induced hyperthermia. Heatstroke is the most severe form of heat illness, and is of two forms: Classical heatstroke (CHS) occurs after exposure to extreme environmental conditions; for example, in heat waves. Exertional heatstroke (EHS) may occur after strenuous physical activity, and may be seen in endurance athletes, the military, and others. Hyperthermia is associated with a number of pharmaceutical agents. Immediate cooling is the mainstay of treatment, with a delay in a reduction in the temperature associated with increased mortality [2]. In CHS, cooling to below 38.9 °C within 60 min is associated with a trend towards improved survival [3]. Further discussion on treatment is outside the scope of this review and is the subject of a separate review in this series.

The neurological and cognitive sequelae of elevated temperature on the brain may be marked during the initial event and also persist to a much later stage or remain permanent despite fever resolution. In this article, we briefly review the cognitive and neurological effects of hyperthermia on the brain, and examine some of the proposed mechanisms by which cerebral damage is caused.

## Clinical patterns

Most patients recover well after a period of hyperthermia, but patients exposed to higher temperatures for longer periods of time are more at risk of complications, which in extreme cases may progress to multi-organ failure and death. The risk may be significant; heatstroke, for example, is associated with a mortality rate of 40 % [4] to 64 % [2].

Patients who become acutely hyperthermic often display signs of neurological dysfunction. The neurological injury may manifest in several ways, including cognitive dysfunction, agitation, seizures, unsteadiness, or disturbance of consciousness from lethargy to coma. Neurological dysfunction in heatstroke is well described, and has been recognised since at least Roman times [5]. Indeed, the presence of neurological dysfunction is required for the diagnosis of EHS in combination with hyperthermia. Cognitive dysfunction also happens quickly with hyperthermia and may take various forms.

### Cognitive dysfunction

Cognition refers to mental abilities and processes, and includes memory, knowledge, attention, reasoning, problem solving, and comprehension. The precise anatomical location of each aspect is not known, and probably involves connections across numerous parts of the brain [6] including the cerebellum [7].

Hyperthermia, even if mild and only occurring for a short period, may cause cognitive impairment. In a few cases, this may be permanent. Hyperthermia has been shown to adversely affect attention [8], memory [9], and processing of information [10] acutely. Some of the cognitive processes may be affected by hyperthermia more than others. Short-term memory processing, for example, may be more affected than attentional processes [11].

Cognitive impairment may occur after exposure to more modest temperatures, and after shorter periods of time, than has previously been recognised. One study of induced hyperthermia in healthy volunteers showed that memory was impaired at a core temperature of only 38.8 °C compared with normothermia [12]. Artificially induced hyperthermia may induce cognitive impairment after only 1 to 2 h of temperature elevation [10, 13]. Cognitive changes may not occur immediately at the time of hyperthermic insult, but instead develop a short time (60–120 min) after the cessation of the insult [13].

Functional neuroimaging supports there being a large number of pathways and connections in cognitive pathways, with many of these being affected acutely in hyperthermia. In general, connections appear to be increased around the limbic system [14], consistent with the observed changes in memory and learning ability. The dorsolateral prefrontal cortex (involved in executive functions—for example, memory, cognition, and reasoning), and the intraparietal sulcus (involved in processing and memory) also show increased activity in acute cerebral hyperthermia [15]. Conversely, connections in other parts of the brain, including the temporal, frontal and occipital lobes, appear to be reduced in acute hyperthermia [14].

Hyperthermia-induced changes in short-term memory formation can also be detected using electroencephalography. The electrical response of the brain to a specific cognitive or sensory event is termed an ‘event-related potential’ (ERP). If the brain is subjected to a repeated identical sound, and then an alternate sound is introduced, the ERP is altered, termed ‘mismatch negativity’ (MMN). MMN has validity in studies into auditory memory formation. Subjects exposed to hyperthermia for as little as 1 h show significant decline in MMN compared with a control group [10], consistent with clinical observations of a decline in short-term memory.

However, it is not clear whether hyperthermia per se is causing these acute cognitive changes, or if the heat is affecting changes through or in combination with other mechanisms. If hyperthermic subjects are kept well hydrated, cognitive impairment may be minimal, suggesting that some of the cognitive dysfunction is due to dehydration. Body weight loss of 1.0–1.5 kg has been reported in studies of cognitive change [13], suggesting dehydration, and it is not clear what effect this has on the cognitive function.

While advancing age is associated with a reduced cognitive baseline, hyperthermia may not reduce function proportionately more than in younger people [16]. While baseline accuracy and ability in attention, memory, and reaction was lower in older volunteers than younger, hyperthermia did not alter these reactions differently in the two groups.

In the majority of cases, patients recover fully from the acute cognitive dysfunction. Some, however, are left with persistent changes in attention, memory, or personality [17]. These may be mild, or severe, up to and including severe global dementia. They have been reported after CHS [18], EHS [19], and drug-induced hyperthermia [20, 21].

### Neurological effects

Neurological manifestations of hyperthermia have been divided into three groups according to the time sequence in which they occur: the acute stage, the convalescent period, and the period of permanent deficits. The magnitude and the duration of the hyperthermia are thought to influence the development of neurological manifestations. In addition, genotypic and phenotypic differences in the physiological response to hyperthermia (for example, in the inflammatory response, or the induction of thermoprotective mechanisms) may also affect an individual’s risk of developing neurological deficits.

#### Acute deficits

Acute neurological deficits have been described after hyperthermia from a number of causes, including heat illness and drugs. Deficits are well recognised after CHS—in the acute phase, many patients have disturbance of consciousness up to and including deep coma. Of 87 patients with CHS after a Mecca pilgrimage, all had a reduced level of consciousness, and 25 (29 %) were deeply comatose [22]. Constricted pupils were reported in all cases in this series, with 17 (20 %) showing automatisms, including chewing, swallowing, and lip smacking. Delirium, lethargy, disorientation, seizures, hypertonia, and hypotonia are also described [23].

Acute neurological damage after drug-induced hyperthermia has been reported to result from malignant hyperthermia (MH) [24] and neuroleptic malignant syndrome (NMS) [20]. Most survivors of NMS recover completely, with a mean recovery time of 7–11 days [20]; the incidence of long-term sequelae has been reported at 3.33 % [21].

#### Persisting deficits

Neurological deficits that persist after the acute phase are well described. The incidence is difficult to discern; of 87 patients who developed CHS during the same Mecca pilgrimage, 75 (87 %) made a full recovery, 10 patients (11 %) died, and two (3 % of the survivors) recovered but developed pancerebellar syndrome [22]. Of 36 patients with EHS, eight (22 %) died, two (7 % of the survivors) had cognitive impairment, and a further two (7 %) had neurological signs, one with paraplegia, and one with cerebellar disturbance [17].

However, the mortality and morbidity after a severe episode may be more significant than these data suggest; if patients require admission to an ICU for management of hyperthermia, the mortality may approach 50 % to 65 % [2, 25], and persistent neurological effects may affect 50 % of survivors [25]. Early deaths are usually from multi-organ failure but, in one study, 50 % of the deaths were comatose or tetraplegic [2]. In one series of patients admitted to ICU with CHS after a heatwave, 33 % had significant neurological impairment, and 33 % of patients had mild impairment at discharge. Only 24 % of patients had no neurological impairment [23].

Persistent neurological deficits have been reported after CHS (the majority) and EHS. Cases after drug-induced hyperthermia are also reported, but are much rarer. Drugs known to cause hyperthermia and persistent neurological deficits include those responsible for neuroleptic malignant syndrome [20, 26–29], and the serotonin syndrome [30], and Chinese herbal medications [31].

Cerebellar dysfunction is by far the predominant clinical picture in cases of persistent neurological dysfunction [17, 18, 22, 25, 32–35]. Ataxia, dysarthria, and co-ordination problems are common; nystagmus is more rarely reported [32]. Of the five reported cases of persistent neurological dysfunction after NMS, all showed cerebellar signs [20, 26–29]. Less common is damage to the cerebral cortex [34, 36], brain stem [37], spinal cord [17], and the peripheral nervous system [25]. Frontal dysfunction is rare, but has been reported. Basal ganglia dysfunction is reported after heatstroke [34], and is well recognised after NMS [21, 38]; the latter may represent the effects of treatment in some patients rather than damage from hyperthermia. Clinical features are usually bilateral. Neurological dysfunction may be profound; a persistent vegetative state has been reported [39]. Patients may show signs of improvement over weeks or months [36] but, in some cases, it may persist for many months or years [18]. Recovery may be minimal or absent [33]. In the vast majority of these reported cases, a core body temperature of 40 °C or higher is recorded.

#### Hyperthermia after brain damage

Fever after brain injury is common, affecting over 70 % of patients after a traumatic brain injury (TBI) [40] and in more than 50 % of patients after a subarachnoid haemorrhage (SAH) [41]. In a proportion, the fever is related to the neurological injury rather than infection; non-infective fever may account for up to one third of cases of fever after a stroke and may affect over a third of patients after TBI [42]. Development of a fever is associated with poor outcome; a fever worsens functional disability and mortality after a stroke [43] and SAH [44].

Adverse effects occur with only small changes in temperature; variations in the brain temperature of just 1 °C can critically affect the extent of secondary brain injury after a primary insult [43]. Mortality after a stroke has been shown to increase at temperatures above 37.9 °C [45], and with a temperature of above 38 °C following TBI [46].

## Radiological and pathological findings

Various radiological findings have been described on magnetic resonance imaging (MRI) after heatstroke, including haemorrhage, oedematous changes, ischaemia, encephalitis, and atrophic changes [37, 47], suggesting a number of pathological processes. Lesions have been observed throughout the central nervous system (CNS), including the brain stem, cerebellum, hippocampus, external capsule, and cerebrum [37, 47, 48]. Haemorrhage on MRI may represent a poorer prognosis—of eight patients in one follow-up study, three patients had evidence of haemorrhage on MRI imaging, and all three died. The remaining five showed no haemorrhage, and all survived [47].

Radiological lesions are often bilateral [37, 48], in keeping with the clinical findings. However, clinical features and radiological signs may correlate poorly. Clinical signs may improve and resolve, despite progressive or non-resolving radiological signs [34]. Radiological signs may improve despite little clinical improvement [36], or signs may deteriorate with minimal clinical change and progression to atrophy may occur [33, 39]. Imaging may show defects in areas of the brain without clinical deficits [36] or may be normal, despite profound clinical deficits [32]. Deficits on imaging may develop late; imaging may be normal on presentation, and abnormalities only evident on repeat imaging. MRI is probably more sensitive than computed tomography.

Pathological changes are observed on standard T2-weighted and FLAIR MRI. Diffusion-weighted imaging (DWI), sensitive for ischaemic changes, and susceptibility-weighted imaging (SWI), sensitive for haemorrhage, may be particularly useful in detecting heatstroke-induced changes [37].

Neuropathological studies of humans with hyperthermic damage are rare, but cerebellar damage is frequently observed, in keeping with the clinical syndrome. In one reported study of patients with CHS, the Purkinje cells displayed the most marked thermal damage [49]. Purkinje cells are found predominantly in the cerebellum, and regulate motor function. Almost complete loss of the Purkinje cells may be seen when death occurs after more than 24 h [50]. Neuronal loss may be seen in other parts of the brain and be replaced by glial cells. Oedematous and haemorrhagic change is also reported [50]. In one case series of eight deaths after EHS, reports of six autopsies showed petechial haemorrhages in a number of locations in five brains, including the meninges, ventricles, cerebellum, and hypothalamus; an intracranial bleed in two, and venous congestion in three. Four of the brains had cerebral oedema, and Purkinje cell degeneration was found in four [17].

NMS may produce similar neuropathological findings; cerebellar damage may be the most marked, with Purkinje cell loss and replacement by Bergmann’s glia. Infiltration by macrophages, and axonal and myelin degeneration also occurs [26]. In a young patient who died after developing MH following an appendicectomy, cerebellar damage predominated, with oedema and herniation [24]. The reason for this selective thermosensitivity is not clear.

## Mechanism of cerebral damage

**Fig. 1:**
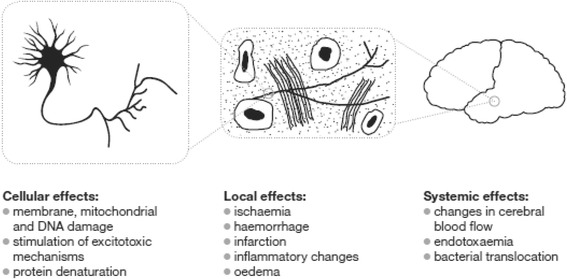
Proposed mechanisms of neuronal damage in hyperthermia

The mechanism by which cellular and cerebral injury and death occurs is not yet fully defined, but is probably multi-factorial. It may be grouped into three broad areas (Fig. 1); some of these are explored briefly below.

- Cellular effects: membrane, mitochondrial and DNA damage, stimulation of excitotoxic mechanisms, protein denaturation
- Local effects: ischaemia, inflammatory changes, oedema, cytokine release, vascular damage
- Systemic effects: changes in cerebral blood flow, endotoxaemia, bacterial translocation through a dysfunctional gastrointestinal tract.

### Cellular effects of hyperthermia

#### Cellular denaturing

Hyperthermia has a number of deleterious effects on neuronal structure and function, including disruption of electrochemical depolarisation, trans-membrane ionic transport and disruption of cellular signalling mechanisms and mitochondrial function [51]. The most temperature-sensitive cellular elements of neural cells are mitochondrial and plasma membranes; irreversible alterations to protein structure appear to occur at temperatures above 40 °C [52]. Protein denaturing may occur even at normothermia [51], suggesting that repair mechanisms are sufficient under normal conditions. At increasing temperatures, the rate of damage increases; while this process may be reversible, denatured proteins ultimately form aggregates which disrupt normal cellular function and prevent replication, and ultimately cell death. In addition, hyperthermia potentiates neuronal damage caused by other toxic insults, for example hypoxia and ischaemia [52].

#### Neuronal death

Thermal stress may result in necrotic cell death, or apoptosis, i.e. stimulation of programmed cell death. Cell death, depending on the cell line, begins to occur at temperatures above 40–41 °C and increases in an exponential manner as temperature exposure time increases [53]. Apoptosis is probably more of a risk in rapidly developing cells than in the adult cerebrum, and may only become a threat at significantly elevated temperatures [54]. Protein denaturing is considered to be the most intimately related factor to cell death [55], but the precise mechanism varies between cell types [55]. In some cells, alterations to signalling pathways are implicated. Caspases are cysteine proteases thought to be intimately involved in the regulation of apoptosis and other cellular regulatory processes. Caspase-mediated cell death may be stimulated by a number of mechanisms; caspase inhibitors appear to prolong neuronal survival after hyperthermic stress [53].

Cell death, when it occurs, may not be immediate. The temperature may be high enough to produce cell swelling and necrotic death during or shortly after the heat stress. More moderate hyperthermia may not produce such signs immediately, with cell death occurring over the next few days [51].

#### Excitotoxicity

Excitotoxicity is the pathological process by which neurones are damaged or killed by excessive exposure to excitatory neurotransmitters—for example, to glutamate and glycine—which may play a role in damage after a traumatic brain injury and neurodegenerative disorders of the CNS, including Alzheimer’s disease and multiple sclerosis. Increased exposure to excitatory neurotransmitters renders neurones susceptible to calcium influx, which may reduce ATP production, alter electrochemical gradients, and stimulate caspase-dependent apoptosis. After an acute ischaemic stroke, glutamate and glycine levels are significantly higher in hyperthermic patients than in normothermia, and the degree of hyperthermia has further been found to be related to infarct size [56]. Glycine mostly functions as an inhibitory neurotransmitter within the CNS, but is required with glutamate to co-activate the NMDA receptor.

#### Protection by heat shock proteins

Heat shock proteins (HSP) are a family of proteins that are produced by cells in response to exposure to stressful conditions, and are protective against a wide variety of noxious stimuli, including ischaemia and hyperthermia. On exposure of the CNS to hyperthermia, HSP expression is predominantly in glial and Purkinje cells. However, as noted above, the Purkinje cells appear to be the most at risk for damage, and the relationship between HSP expression and this selective thermosensitivity is not clear. Many of the sensory and motor neurones of the CNS show constitutive expression of HSP 27, with further inducible expression with noxious stimuli. HSP 70 has little or no constitutive expression [57]; expression of HSP 70 in Purkinje cells may be delayed by several hours, which may render the cells at risk in the immediate phases. Other workers suggest that, while HSP confers some protection, it is also a marker of a cell at risk of damage from thermal insult [52]. HSPs are expressed in response to the insult, and their effect may depend on their location: intracellularly located HSPs have a protective role, including correcting misfolded proteins, preventing protein aggregation, transport of proteins, and supporting antigen processing and presentation, and limiting apoptosis. In contrast, membrane-bound or extracellular HSPs may be immunostimulatory, and appear to induce cytokine release or provide recognition sites for natural killer cells; HSPs may have pro-apoptotic, as well as anti-apoptotic action [58, 59]. Finally, cell death by apoptosis may occur through different mechanisms, and recent work suggests that HSP may not provide protection from all these mechanisms [55].

Thermosensitivity of specific cell lines may be explained by selective expression of other stress-related proteins. More recent work has isolated heme oxygenase (HO)-1, the expression of which is predominantly in the Purkinje cells. Induction of HO-1 aggravates heat shock injury in cerebellar cells [60].

#### Changes in cell signalling

Recent work suggests that cellular function is altered by hyperthermia-induced phosphorylation of members of the kinase family, critically involved with regulation of cellular metabolic pathways, in addition to caspase-regulated apoptosis.

The c-Jun NH2-terminal protein kinase (JNK) plays important roles in a broad range of physiological processes. JNK 2 and JNK 3 are involved in the regulation of cell differentiation and development, including the induction of apoptosis in response to neuronal stress. Heat stress alters the function of JNK by alteration in its phosphylation state [61].

Hyperthermia-induced cognitive dysfunction which persists may be partly explained by alterations in cell signalling. Calcium/calmodulin-dependent protein kinase II (CaMKII) is involved in many signalling cascades and is thought to be an important mediator of learning and memory. Exposure to hyperthermia sufficient to cause seizure in neonatal rats showed significant deficits in spatial learning and memory. When analysed as adults, phosphorylation of CaMKIIα Thr(286) was reduced significantly, but the phosphorylation of CaMKIIα Thr(305) was increased significantly, despite the initial thermal insult occurring as neonates [62].

### Local effects of hyperthermia

#### Cytokine induction and the inflammatory response

The expression of a large number of pro- and anti-inflammatory cytokines changes during the acute and recovery phases of a hyperthermic insult. While the precise expression varies depending upon the cause of the hyperthermia, the pro-inflammatory cytokines interleukin (IL)-1 and tumour necrosis factor (TNF)-α and the anti-inflammatory cytokines IL-1RA, IL-10 and soluble TNF receptors are commonly raised in hyperthermia, with changes in IL-6 concentration being particularly related to outcome [63]. Indeed, reduction of the pro-inflammatory response may be associated with a better prognosis.

### Systemic effects of hyperthermia

#### Blood–brain barrier disruption and the development of oedema

The blood–brain barrier (BBB) under normal conditions is a very selective barrier of tight endothelial cells, preventing the movement of large or hydrophilic molecules, or toxic substances, into the brain. The permeability of the BBB is highly temperature-dependent, allowing significantly increased transport of substances at temperatures above 38–39 °C, increasing further at higher temperatures [64]. This increased permeability is considered by some to be the predominant factor in the development of cerebral oedema in hyperthermic states. The temperature at which cerebral oedema develops corresponds well to that at which disruption to the BBB occurs: cerebral oedema has been reported in patients dying of heat-related illness with a core temperature at death of 39 °C [65]. Rats, with a similar core temperature to humans, develop cerebral oedema and a more permeable BBB at temperatures above 38.5–39 °C [64].

#### Cerebral blood flow and metabolism

Cerebral oxygen and glucose consumption generally increase in hyperthermic states, but the precise relationship with temperature is unclear, and there is considerable regional variability [66]. With modest increases in core temperature, cerebral metabolic rate increases in some areas, but decreases in others. In more extremes of hyperthermia, mitochondrial oxygen metabolism may not be increased beyond that experienced at normothermia, and beyond 40 °C may then decrease [66]. This may imply either impaired uptake of mitochondrial oxygen at hyperthermic temperatures, but in the absence of a raised lactate may indicate a reduction in cerebral metabolic activity at increased temperatures, and thereby account for the cognitive and neurological signs and symptoms.

Similarly, changes in cerebral blood flow (CBF) are incompletely understood. Animal studies suggest that CBF may increase by up to 24 % for every 1 °C increase in temperature [67], but at temperatures above 40–41 °C regional CBF may fall to baseline or below [66, 68]. At temperatures above 40 °C in humans, coupling of cerebral flow–pressure becomes deranged. In one study of patients undergoing therapeutic hyperthermia for hepatitis C infection, raising the core temperature to 41.8 °C was associated with an increase in CBF velocity of 1.5- to 2-fold, independent of the arterial blood pressure [69]. This impairment of flow–pressure coupling may aggravate vascular engorgement and intracranial hypertension, and therefore cerebral oedema [66]. Disruption of autoregulation may further disrupt BBB integrity, rendering the brain more at risk from systemic insults.

#### Gut translocation

Systemic hyperthermia increases the permeability of the gastrointestinal (GI) tract, and increases the rate of gut bacterial translocation. Blood flow to the GI tract is reduced, and hyperthermia damages cell membranes, denatures proteins, and enhances production of free radicals. The overall result is loss of GI barrier integrity and the potential for GI-derived endotoxins to enter the internal environment. Endotoxaemia also initiates the release of pro-inflammatory cytokines that can produce a systemic inflammatory cascade and multiple organ damage. Hyperthermia reduces the integrity of the BBB [64], raising the possibility that some of the neurological dysfunction is related to GI bacterial or endotoxin translocation. In an animal model of heatstroke, antibiotics improve survival [70].

#### Heavy metal toxicity

Metals are implicated in a number of neurological clinical syndromes in normothermic conditions. Metal may be responsible for activation of the free radical-induced cellular damage in tissues other than the brain in hyperthermia [71], and damage to the BBB, for example, by hyperthermia may exacerbate metal-induced neurotoxicity. Nanoparticles derived from metals induced brain dysfunction in normal animals, but under hyperthermic conditions the cognitive and motor deficits were much more marked [72].

## Conclusions

Hyperthermia is a common insult to the CNS. A variety of neurocognitive effects are reported, which, in some cases, may persist after the acute insult. Changes take place at lower temperatures, following shorter periods of time, than may have been previously appreciated. In long-term cases, cerebellar damage predominates, thought to be a result of the sensitivity of the Purkinje cells to thermal damage. A core temperature of 40 °C or above is associated with either long-term or permanent neurological damage, consistent with the cellular changes and cell death occurring above this temperature. Recovery may be minimal; the mainstay of treatment therefore remains immediate recognition and cooling to minimise complications.

A variety of histopathological and neuroradiological changes are reported after acute hyperthermia; however, these changes appear to show poor correlation with the clinical picture overall.

The mechanism of the cerebral damage remains unclear, but is likely to be a combination of direct cytotoxic damage from the hyperthermia and indirect systemic effects inhibiting neuronal function.

It is not clear which patients are most at risk, but it is likely that the individual’s physiological response, as well as the duration of the hyperthermia, may be important. Further work is needed to clarify how genotypic and phenotypic differences predispose an individual to developing neurological deficits. Current treatment is essentially limited to urgent cooling by physical methods; elucidating the pathogenesis of neurological deficits may allow development of additional treatment. Of interest is the role of the inflammatory response, and bacterial and endotoxin translocation from the GI tract, which may provide novel targets for future treatments to minimise or prevent neurological complications after hyperthermia.

## Abbreviations

BBB, blood–brain barrier; CaMKII, calcium/calmodulin-dependent protein kinase II; CBF, cerebral blood flow; CHS, classical heatstroke; CNS, central nervous system; DWI, diffusion-weighted imaging; EHS, exertional heatstroke; ERP, event-related potential; GI, gastrointestinal; HO, heme oxygenase; HSP, heat shock protein; ICU, intensive care unit; IL, interleukin; JNK, c-Jun NH2-terminal protein kinase; MH, malignant hyperthermia; MMN, mismatch negativity; MRI, magnetic resonance imaging; NMS, neuroleptic malignant syndrome; SAH, subarachnoid haemorrhage; SWI, susceptibility-weighted imaging; TBI, traumatic brain injury; TNF, tumour necrosis factor
